# Anti-Biofilm Inhibitory Synergistic Effects of Combinations of Essential Oils and Antibiotics

**DOI:** 10.3390/antibiotics9100637

**Published:** 2020-09-24

**Authors:** Antonio Rosato, Sabina Sblano, Lara Salvagno, Alessia Carocci, Maria Lisa Clodoveo, Filomena Corbo, Giuseppe Fracchiolla

**Affiliations:** 1 Department of Pharmacy—Drug Sciences, University of Bari “Aldo Moro”, via E. Orabona, 4-70125 Bari, Italy; sblano.sabina@gmail.com (S.S.); l.salvagno@studenti.uniba.it (L.S.); alessia.carocci@uniba.it (A.C.); filomena.corbo@uniba.it (F.C.); giuseppe.fracchiolla@uniba.it (G.F.); 2 School of Medicine: Interdisciplinary Department of Medicine, University of Bari “Aldo Moro”, piazza G. Cesare, 11-70124 Bari, Italy; marialisa.clodoveo@uniba.it

**Keywords:** synergism, anti-biofilm, antibiotic-resistance, *Mentha piperita*, *Cinnamomun zeylanicum*, *Origanum vulgare*, *Thymus vulgaris*, norfloxacin, oxacillin, gentamicin

## Abstract

In recent years, the increase of bacteria antibiotic- resistance has been a severe problem for public health. A useful solution could be to join some phytochemicals naturally present in essential oils (EOs) to the existing antibiotics, with the aim to increase their efficacy in therapies. According to in vitro studies, EOs and their components could show such effects. Among them, we studied the activity of *Cinnammonum zeylanicum*, *Mentha piperita*, *Origanum vulgare*, and *Thymus vulgaris* EOs on bacterial biofilm and their synergism when used in association with some common antibiotics such as norfloxacin, oxacillin, and gentamicin. The chemical composition of EOs was determined using gas chromatography (GC) coupled with mass spectrometry (MS) techniques. The EOs drug efficacy was evaluated on four different strains of Gram-positive bacteria forming biofilms. The synergistic effects were tested through the chequerboard microdilution method. The association EOs-antibiotics showed a strong destruction of the biofilm growth of the four bacterial species considered. The interaction of norfloxacin with EOs was the most effective in all the tested combinations against the strains object of this study. These preliminary results suggest the formulation of a new generation of antimicrobial agents based on a combination of antimicrobial compounds with different origin.

## 1. Introduction

The dramatic increase in anti-microbic resistance (AMR) for pathogenic bacteria represents a serious problem for human health [1]. It occurs by the mechanism of selective pressure of the antibiotic. This mechanism destroys all the bacteria of a certain species, allowing the survival to all those species that have a mutation that makes them resist to the action of the drug. The consequence is the appearance of bacterial sub-population resistant to the action of antibiotics promoting super-infections that are more difficult to treat pharmacologically [2].

Despite the effort aimed at curbing resistance to antibiotics, the situation is constantly worsening [2,3]. In fact, AMR leads to higher medical cost, prolonged hospital stays and increased mortality [2,3,4]. For this reason, a solution is to hinder the uncontrolled use of antibiotics and search for new ones [3]. Moreover, it is essential to decrease the use of the antibiotic in the veterinary and agricultural fields and could be interesting to exploit synergistic interactions with natural products such as plant products [5].

Recently, the idea that essential oils (EOs) can help to limit antibiotic resistance has been accepted by the scientific community [3,6,7]. EOs are extracted from various plants through various techniques including fermentation, enfleurage, extraction, and steam distillation [7,8], the latter being the reported technique of choice in the Pharmacopoeia [9]. EOs show antibiotic, antifungal, insecticidal, and antiviral activities. Although their therapeutic properties have been known for more than 2000 years, in the last 200 years, these properties have gone into the background to give more importance to the aspect related to the fragrance and the food additives, aspects most requested by the market [3].

In the literature, there are several data about the EOs activity as antibiotics, and their chemical composition seems to play a pivotal role in their application as antibacterial agents [3,7,9].

The EOs mechanism of action has been extensively reported in the literature and concerns the breakdown of bacterial cell wall, although the effect on the destruction of enzymes or membrane proteins or the spillage of cellular content after cytoplasmic membrane breakage are also possible [3,10,11]. According to these data, synergistic effects between antibiotics and EOs against bacteria can occur [7]. This aspect is important because it will help to reduce the use and the dosage of antibiotics in therapies, decreasing both, the antibiotic resistance and side effects [3,7,9].

Several studies report that whole EOs have an antimicrobial activity stronger than their major components individually tested [3]. These evidences show that also the minor components present in EOs can play an important role in the antimicrobial activity and the synergistic effect [3,7,9]. On the basis of this efforts, we planned to evaluate the synergistic effect of the whole *Mentha piperita*, *Cinnamomun zeylanicum*, *Origanum vulgare*, and *Thymus vulgaris* EOs with norfloxacin, oxacillin and gentamicin against four bacterial strains that form biofilm. 

## 2. Results

### 2.1. EOs Chemical Composition

EOs were analyzed by gas chromatography hyphenated with mass spectrometry (GC/MS) technique. Chemical composition of all the EOs was reported in Table 1. For each EO several compounds were identified that are mainly represented by sesquiterpene hydrocarbons, oxygenated monoterpenes, monoterpene hydrocarbons, oxygenated hydrocarbons, diterpene hydrocarbons, and oxygenated diterpenes [12].

About 97% of *Cinnamomun zeylanicum* EO was identified [18]. The major compounds in the *Cinnamomun zeylanicum* EO were *E*-cinnamaldehyde (78.07%), *o*-methoxy cinnamaldehyde, (11.32%) and *E*-cinnamic acid (2.97%).

The major components of pure *Origanum vulgare* EO [19] were thymol (59.25%), carvacrol (25.09%), *p*-cymene (5.09%), (E)-β-caryophyllene (1.8%), and caryophillene oxide (1.66%). The main components of pure *Thymus vulgaris* EO [20], were (D, L)-isoborneol (26.34%), (E)-β-terpineol (12.28%), thymol (11.35%), (E)-β-caryophyllene (7.37%), camphene (6.79%), *α*-pinene (5.35%), p-cymene (5.09%), and α-terpinolene (5.03%), while eucaliptol, limolene, γ-terpinene, and bornyl acetate were less than 2% each one. The other minor components were reported in Table 1 in the line described as Others and were in traces. The identified compound, instead, are respectively 97.69% for *Origanum vulgare* EO and 96.48% for *Thymus vulgaris* EO.

Pure *Mentha piperita* EO was identified for 97.58% of its composition [21,22]. Menthol is the major component being the 67.98% of the whole EO followed by 1-menthone, representing the 17.87% of the mixture. Other compounds are present in traces and correspond to 2.42%.

### 2.2. Antibacterial Activity

In this research, we used different antibiotics in association with EOs in order to inhibit the bacteria biofilm growth of four Gram-positive bacteria strains producing biofilm. The effects of these associations on the destruction of the bacterial biofilm were reported in Table 2, Table 3 and Table 4 as percentage values. The FIC index (FICI), a parameter that studies the synergism of two compounds, was reported too. Considering the association between EOs and antibiotics, the percentage of destruction was in the range of 50.3–72.1% for gentamicin, 47.7–66.9% for norfloxacin and 45.2–67.6% for oxacillin. It is interesting to notice that the amounts of antibiotics used to obtain the reduction of biofilm was remarkably decreased when combined with EOs. It is possible to notice how reduction in concentrations for antibiotics ranged from 25 to 33 times for gentamicin. Comparable results were observed in association with oxacillin (from 18 to 32 times) and norfloxacin (from 18 to 25 times). FICI ranged from 0.08 to 0.23 for oxacillin association, from 0.08 to 0.16 for gentamicin and from 0.08 to 0.23 for norfloxacin in association with EOs. In the whole experimentation, these aspects confirmed the existence of a strong synergism with a FICI value lower than the limit value 0.5 [23].

## 3. Discussion

These promising results allow us to confirm the synergistic effects between the essential oils and antibiotics studied. In fact, the data obtained clearly show a significant reduction in the concentration of antibiotics when used in association with essential oils. In particular, this work emphasizes the efficacy of *Cinnamomum zeylanicum* EO associated with antibiotics, when it is compared with other common EOs studied [21,22,24,25].

Table 2, Table 3 and Table 4 report data on sMIC_50_ and synergism. The sMIC_50_ value of gentamicin in association with *Cinnamomum zeylanicum* is reduced from 128 µg/mL to 3.99 g/mL for *Enterococcus faecalis* ATCC 29212 and *Staphylococcus aureus* Ig22. These results underlined the large reduction of the quantities of EOs employed to attain the association with respect to the quantity of the EO used alone to inhibit the strain. Another indicative example is the biofilm destruction of *Staphylococcus aureus* ATCC 29213. The use of the antibiotic alone requires 512 µg/mL of gentamicin, this quantity decreased 33 times when it was used in association with *Mentha piperita* EO that is, in turn, reduced 20 times. Table 2, Table 3 and Table 4 illustrate these examples for the tested strains, as the association *Cinnamomum zeylanicum* EO-oxacillin, the antibiotic quantity is reduced from 64 µg/mL to 2 µg/mL. These effects are usual for all the association we considered during the experiments. For oxacillin the quantity employed against *Staphylococcus aureus* Ig22 alone ranged from 64 µg/mL to 512 µg/mL to obtain the destruction at least of 50% of the biofilm growth, while the quantities of antibiotics used in association ranged from 2 µg/mL to 16 µg/mL. A further example of 50.4 mg of *Cinnamomum zeylanicum* EO are necessary to inhibit *Enterococcus faecalis* ATCC 29212 biofilm, when the EO is combined with antibiotic oxacillin this quantity is dramatically reduced from 50.4 mg/mL to 2.5 mg/mL. If we take into consideration this strain the association EO- oxacillin produces a large destruction of biofilm equal to 56.6 ± 1.00%, with limited quantities of the two compounds, antibiotic (66.0%) or EO (52.5%) that caused a lower effect of destruction with more large quantities [26].

Results about synergism are reports in Table 2, Table 3 and Table 4 as the percentages of biofilm destruction obtained with a very limited quantity of the component of the association. Average values of antibiotics reduction were from 8.33 to 30 times, ranging from 5.0 to 20.54 times with respect to the quantities of the two components used alone. The associations *Thymus vulgaris* and *Cinnamomum zeylanicum* gave the best results with a better value of percentage of destruction. The FICI of the associations EOs-antibiotics showed a very strong synergistic interaction for all the tested bacterial strains. As detailed above, the combination of EOs with antimicrobials is the origin of a significant reduction in the concentration of gentamicin and the other two antibiotics—oxacillin and norfloxacin—used to inhibit the biofilms.

## 4. Material and Methods

### 4.1. Materials

The pure EOs were provided by Erbe Nobili srl (Corato, Bari, Italy). They were stored in a brown glass bottle at 0–4 °C until the testing analysis or microbiological assays.

All the solvents and the analytical standard were in HPLC grade and were purchased from Sigma-Aldrich S.r.l. (Milan, Italy). Filters were supplied by Agilent Technologies Italia S.p.a (Milan, Italy).

All the antibiotics used and the crystal violet reagent were purchased from Sigma-Aldrich S.r.l (Milan, Italy). The colture media used are Triptic Soy Broth with 10–25% glycerol (Oxoid, Italy) solution, Mueller Hinton Broth (Oxoid, Italy) and Mueller Hinton agar (Oxoid, Italy).

### 4.2. Methods

#### 4.2.1. Gas Chromatography and Mass Spectrometry

Each sample was prepared diluting 1:10 the pure EO in Ethyl Acetate. All EOs sample were analyzed by GC technique using an Agilent 6890N Gas Chromatograph coupled with a 5973N MSD HP ChemStation, equipped with autosampler. The gas chromatograph is equipped with a split-splitless injector and a HP-5 MS (5% phenylmethylpolysiloxane, 30 m, 0.25 mm i.d., 0.1 μm film thickness; J & W Scientific, Folsom) capillary column. Gas chromatography conditions were 5 min at 40 °C, then 4 °C/min to 280 °C, held for 15 min, for a total run of 45 min. The injector and the detector were maintained at a temperature of 280 °C. The inert gas, used as carrier, was helium (He) at a flow rate of 1.0 mL/min. The injected volume in each analysis was 1 μL. The MS conditions were the following: The ionization voltage was 70 eV, while the ion source temperature was 220 °C and the acquisition range was 35–360 amu.

The percent composition of volatile compounds of EOs was calculated comparing areas of identified compound. The qualitative analysis was based on the percent area of each peak of the sample compounds. For EOs components identification was used the comparison with authentic standards available in the authors laboratory whenever possible. Otherwise, the correspondence of Linear Retention Indices (LRIs) [20], and mass spectra (MS) with respect to those reported in commercial libraries as NIST 2011 (USA National Institute of Science and Technology software, 2011) [22], and ADAMS (4th Ed.) [21], was considered reliable in the peak assignment. Semi-quantification of EO components was carried out by peak area normalization considering the same GC response of the detector towards all volatile constituents. Table 1 shows the chemical composition of the analyzed EOs.

#### 4.2.2. Microbial Strains and Antimicrobial Testing

Four bacterial strains from American Type Culture Collection (ATCC, Rockville, MD, USA) and clinical isolates were used as controls to test the anti-biofilm properties of the EO and synergistic effect in combination with antibiotic drugs, *Staphylococcus aureus ATCC 29213, Enterococcus faecalis ATCC 29212, Staphylococcus epidermidis IG4, Staphylococcus aureus IG22.* The last two strains were isolates from Department of Biomedical Science and Oncology—University of Bari. The isolates were identified by assimilation profiles using the biochemical tests performed with the commercial system API^®^ (bioMérieux, Marcy l’Ete, Grenoble, France). Stocks were maintained at −80 °C in Triptic soy broth with 10–25% glycerol (Oxoid, Italy) solution. All strains were stored at −20 °C in glycerol stocks and were subcultured on Muller Hinton agar plates (Oxoid, Rodano, Italy) to ensure viability and purity before the beginning of study.

The bacterial species were cultured on Mueller Hinton agar (MHA, Oxoid), and each bacterial suspension was composed of 2–3 colonies of each strain taken from an MHA plate and dissolved in 2 mL of MHB (Mueller Hinton Broth, Sigma-Aldrich, St. Louis, MO, USA) [26].

#### 4.2.3. Biofilm Biomass Measurement and Reduction

The checkerboard procedure was performed to evaluate the synergistic anti biofilm action of EOs in association with the antimicrobial drugs. In brief, 200 µL of a bacteria culture (10^6^ cfu/mL) was added to each well of a microtiter plate and incubated for 24 h at 37 °C by shaking on a rocker table to allow cell attachment and biofilm formation. Afterward, 200 µL of antibiotic was added as positive control, while the negative control was containing only MHB instead of EO-drugs association. Following incubation, the contents of each well were removed, wells were rinsed with sterile PBS 100 µL to remove loosely attached cells and non-adherent and nonviable cells. After incubation, 200 µL of each combination EO drug was added to the wells. The plates were oven dried at 60 °C for 60 min. This step was endorsed by staining the recovered wells with crystal violet 2%. The wells were stained with 150 µL of 2% crystal violet, incubated at room temperature for 50 min. After this step the plates were washed three time with sterilized PBS to remove unabsorbed color, then the wells were destained by adding 100 µL of ethanol. The solution so obtained was transferred to a new plate and the absorbance was measured at OD_625_ using a microplate spectrophotometric reader. Each assay was performed in triplicate. The mean absorbance of each combination EO-drug was determined, the absorbance in control well was subtracted form absorbance reading and percentage of reduction was determined. Percentage of reduction of biofilm obtained with the combinations studied was determined as follows: percentage of biofilm reduction = (OD control well−OD experimental)/(OD control well) × 100 [27].

#### 4.2.4. Microdilution Checkerboard Method

In the combination assays for biofilm, the checkerboard procedure as described by Rosato et al. [21,28] was followed to evaluate the synergistic action of the EOs with selected drugs against biofilms. Four double serial dilutions of the EO were prepared following the same method used to evaluate the MIC described in our previous works [21,22,28]. Dilutions of the EOs were prepared together with a series of double dilutions of the drugs (512–32 µg/mL). This method was used to mix all the antimicrobial compounds dilutions with the appropriate concentrations of EOs so that a series of concentration combinations of the EOs-drugs being considered were obtained. In our experimental protocol, the activity of substance combinations was analyzed by calculating the FICI as follows: FIC of EO plus FIC of drug. Generally, the FICI value was interpreted as (i) a synergistic effect when ≤0.5; (ii) an additive or indifferent effect when >0.5 and <1; and (iii) an antagonistic effect when >1 [23]. The concentrations prepared accounted for 40%, 20%, 10%, and 5% of the MIC value for the EO and 25%, 12.5%, 6.25%, and 3.12% of the MIC value for the antibiotic [23,29].

MIC was defined as the lowest concentration of the mixtures that resulted in no visible growth of the bacterial strains compared to their growth in the control well. MIC determination were performed in four independent assays. MIC data of the antimicrobial compounds and EOs were used to calculate the fractional inhibitory concentration (FIC) that was obtained for each drug by dividing the MIC of the drug, when used in combination, for the MIC of the same drug, when used alone [30].

### 4.3. Statistical Analysis

Every experiment for GC-MS has been replicated three times in three different days. The antimicrobial assays were performed for five times in five different days, giving an amount of 25 replicates. Results obtained were treat calculating trend and then on the three similar values, standard deviation was calculated.

Statistical analysis for microbiological assays (standard deviation, SD) and for chemical determination of structural equation modeling (SEM) was performed using Microsoft Excel, [Microsoft Corporation (2010), Retrieved from https://office.microsoft.com/excel].

## 5. Conclusions

This work is a part of a larger project, which includes the study of the activity of some EOs in combinations with different antibiotics, the results of which appear to be very promising. According to our previous studies reported in the literature [21,22], the presence of oxygenated monoterpenes alcohols and phenols, present on these EOs, give important results in biofilm destruction. Moreover, several researchers have shown that the combination antibiotics and EOs is particularly active in vitro, and it was found to produce a substantial antibiotics MIC reduction against bacterial biofilm whose pharmacological treatment is very difficult. Thus, these compounds could represent a valid option to reduce the use of antibiotics and may help the formulation of new agents for the cure of infections caused by biofilms [31,32].

In conclusion, this work underlines the anti-biofilm effect of some EOs in association with some antibiotics against a set of resistant strains that show a significant antibacterial biofilm. We suppose this work to be a starting point to develop a safer drug formulation that could reduce the health impact of multi-drug resistance to perform further experimental procedures that provide greater details about the mechanism of action of the synergism to confirm these in vitro results. The use of EOs probably will not completely resolve antibiotic resistance problems, but it could play a part in the overall solution to reduce antibiotic use.

## Figures and Tables

**Table 1:** Chemical composition of tested essential oils (EOs).

N	COMPONENTS	LRI	AI	CINNAMOMUN ZEYLANICUM		ORIGANUM VULGARE		THYMUS VULGARIS		MENTHA PIPERITA	
				AREA% ± SEM	SI/MS	AREA% ± SEM	SI/MS	AREA% ± SEM	SI/MS	AREA% ± SEM	SI/MS
1	*n*-propyl acetate	712	712							0.03 ± 0.020	83
2	propanoic acid, ethyl ester	714	714			0.03 ± 0.025	91	0.03 ± 0.010	86	0.04 ± 0.015	86
3	butanoic acid, 2-methyl-, methyl ester	779	780			0.06 ± 0.035	83				
4	α-tricyclene	915	919					0.45 ± 0.25	94		
5	artemisia triene	922	922					0.14 ± 0.10	98		
6	α-thujene	925	926			0.13 ± 0.40	91	0.49 ± 0.50	93		
7	α-pinene	930	934			0.18 ± 0.35	94	5.35 ± 0.99	97	1.45 ± 0.23	97
8	camphene	949	949					6.79 ± 1.01	96		
9	benzaldehyde	956	958	0.91 ± 0.30	97						
10	1-octen-3-ol	980	979			0.55 ± 0.25	90				
11	3-octanone	984	984			0.25 ± 0.10	91				
12	β-myrcene	985	990			0.38 ± 0.20	91	0.36 ± 0.09	83		
13	3-octanol	990	995							0.53 ± 0.04	83
14	2-carene	1020	1021			0.21 ± 0.12	97				
15	eucalyptol	1021	1023			0.11 ± 0.05	97	1.05 ± 0.11	97	0.20 ± 0.02	98
16	*p*-cymene	1024	1024			5.09 ± 0.98	95	5.09 ± 0.99	95		
17	α-terpinolene	1025	1026					5.03 ± 0.85	97		
18	β-phellandrene	1028	1028							0.22 ± 0.01	91
19	limonene	1033	1033					1.16 ± 0.12	97		
20	salicylaldehyde	1040	1041	0.14 ± 0.05	87						
21	γ-terpinene	1063	1060			0.37 ± 0.15	95	1.42 ± 0.20	94		
22	*p*-cymenene	1090	1092					0.36 ± 0.07	96		
23	hydrocinnamic aldehyde	1123	1123	0.19 ± 0.01	93						
24	phenylethyl alcohol	1135	1139	0.45 ± 0.25	91						
25	camphor	1143	1145					1.19 ± 0.9	98		
26	(Ε)-β-terpineol	1145	1145			0.38 ± 0.10	96	12.28 ± 0.99	90		
27	isopulegol	1140	1146							1.35 ± 0.99	98
28	menthone	1148	1150							17.87 ± 1.07	97
29	D,L-isoborneol	1160	1167	0.13 ± 0.01	90	0.55 ± 0.17	97	26.34 ± 1.78	97		
30	menthol	1169	1169							67.98 ± 1.59	91
31	terpinen-4-ol	1174	1174			0.82 ± 0.21	97	2.19 ± 0.98	97		
32	verbenone	1200	1205					0.70 ± 0.04	94		
33	pulegone	1230	1236							0.40 ± 0.01	97
34	carvenone	1248	1252					1.52 ± 0.87	94		
35	*o*-anisaldehyde	1252	1252	0.87 ± 0.08	99						
36	piperitone	1253	1253							0.85 ± 0.30	96
37	(E)-cinnamaldehyde	1266	1266	78.07 ± 1.99	97						
38	bornyl acetate	1285	1287					2.44 ± 0.33	99		
39	*p*-cymen-7-ol	1287	1290			0.11 ± 0.012	70				
40	thymol	1290	1292			59.25 ± 1.80	94	11.35 ± 1.11	99	4.74 ± 0.80	91
41	carvacrol	1304	1304			25.09 ± 1.59	93				
42	durenol	1319	1319			0.14 ± 0.03	78				
43	cubenene	1345	1348	0.35 ± 0.12	95			0.32 ± 0.09	99		
44	α-ylangene	1368	1368	0.17 ± 0.08	80						
45	linalool isobutyrate	1372	1374			0.12 ± 0.06	90				
46	α-copaene	1379	1379					0.13 ± 0.01	99		
47	β-bourbonene	1380	1382					0.13 ± 0.03	97	0.23 ± 0.09	93
48	(E)−β-caryophyllene	1415	1419			1.8 ± 0.28	99	7.81 ± 1.33	99	0.58 ± 0.10	99
49	β-gurjunene	1428	1428					0.14 ± 0.05	90		
50	coumarin	1430	1432	1.00 ± 0.10	95						
51	(E)-cinnamic acid	1455	1457	2.93 ± 0.21	98						
52	alloaromadendrene	1455	1458					0.23 ± 0.04	99		
53	β-farnesene	1459	1459			0.41 ± 0.11	97	0.23 ± 0.07	90		
54	cinnamaldehyde, o-methoxy	1464	1464	11.32 ± 1.50	97						
55	γ-muurolene	1477	1477	0.13 ± 0.09	93			0.37 ± 0.12	97	0.69 ± 0.24	96
56	β-bisabolene	1505	1505	0.17 ± 0.10	86						
57	caryophyllene oxide	1580	1592			1.66 ± 0.59	91				
58	*n*-valeric acid	1722	1720					1.39 ± 0.10	99	0.49 ± 0.05	90
59	benzyl benzoate	1730	1753	0.14 ± 0.09	96						
	% Characterized			96.97		97.69		96.48		97.58	
	Others			3.03		2.31		3.52		2.42	

Linear retention index (LRI) on HP-5MS column was experimentally determined using homologous series of C8-C30 alkanes [13]. Arithmetic index (AI) was taken from Adams 4th Ed. (2011) [14], and/or the NIST 2011 Database [15]. Similarity index/mass spectrum (SI/MS) was compared with data reported on NIST 2011 Database and were determined as reported by Koo et al. [16], and Wan et al. [17]. Database relative percentage values are means of three determinations with a structural equation modeling (SEM) in all cases below 10%.

**Table 2:** Destruction effect of different EOs alone and in combination with gentamicin on mature biofilm.

		EO mg/mL		Gentamicin µg/mL		Synergism			
Strains	Essential Oil	sMIC_50_^a^	%Destr. ± SD ^b^	sMIC_50_^c^	% Destr. ± SD ^d^	AB ug/mL ^e^	EO mg/mL ^f^	AB ± EO%. Destr. ± SD ^g^	FICI
*E. faecalis ATCC 29212*	*Cinnammonum zeylanicum*	50.4	52.5 ± 0.70	128	59.9 ± 0.05	4.0	2.5	64.8 ± 0.05	0.08
*S.aureus Ig22*	*Cinnammonum zeylanicum*	6.3	52.1 ± 0.70	128	52.7 ± 0.40	4.0	0.3	50.3 ± 0.70	0.08
*S.epidermidis IG4*	*Cinnammonum zeylanicum*	12.6	60.0 ± 1.00	64	58.3 ± 0.90	1.9	0.6	63.2 ± 0.80	0.08
*S. aureus ATCC 29213*	*Cinnammonum zeylanicum*	12.6	48.0 ± 1.00	512	52.2 ± 0.70	15.4	0.6	56.7 ± 0.60	0.08
*E. faecalis ATCC 29212*	*Mentha piperita*	11.3	50.0 ± 1.00	128	59.9 ± 0.50	4.0	0.6	55.9 ± 1.00	0.08
*S. aureus Ig22*	*Mentha piperita*	11.3	51.1 ± 0.60	128	52.7 ± 0.60	4.0	0.6	51.2 ± 1.00	0.08
*S. epidermidis IG4*	*Mentha piperita*	45.5	44.8 ± 0.70	64	58.3 ± 0.08	1.9	4.6	51.9 ± 0.50	0.13
*S. aureus ATCC 29213*	*Mentha piperita*	22.8	45.1 ± 1.00	512	52.2 ± 0.60	15.4	1.1	68.7 ± 0.80	0.08
*E. faecalis ATCC 29212*	*Origanum vulgare*	5.5	49.2 ± 0.60	128	59.9 ± 0.80	4.0	0.3	53.2 ± 0.03	0.08
*S. aureus Ig22*	*Origanum vulgare*	11.0	57.3 ± 1.00	128	52.7 ± 1.00	4.0	0.6	63.4 ± 0.10	0.08
*S. epidermidis IG4*	*Origanum vulgare*	5.8	61.9 ± 0.60	64	58.3 ± 0.30	1.9	0.3	50.3 ± 0.60	0.08
*S. aureus ATCC 29213*	*Origanum vulgare*	5.8	56.8 ± 1.00	512	52.2 ± 1.00	30.7	0.3	51.8 ± 0.90	0.11
*E. faecalis ATCC 29212*	*Thymus vulgaris*	21.7	47.8 ± 0.80	128	59.9 ± 1.00	4.0	1.1	51.5 ± 0.60	0.08
*S. aureus Ig22*	*Thymus vulgaris*	43.5	50.5 ± 0.50	128	52.7 ± 0.70	4.0	2.2	72.1 ± 0.70	0.08
*S. epidermidis IG4*	*Thymus vulgaris*	10.9	54.1 ± 0.30	64	58.3 ± 0.10	3.8	1.1	67.0 ± 0.70	0.16
*S. aureus ATCC 29213*	*Thymus vulgaris*	10.9	59.4 ± 0.40	512	52.2 ± 0.80	15.4	0.5	63.0 ± 0.80	0.08

^a^: sMIC50: sessile minimal inhibitory concentration 50; ^b^: %biofilm destruction ± standard deviation; ^c^: concentration of the antibiotic in the combination; ^d^: Concentration of the EO in the combination; ^e^: concentration of antibiotic in the mixture; ^f^: concentration of essential oil in the mixture; ^g^: biofilm combination mixture inhibition rate; FICI: fractional inhibitory concentration; AB: antibiotic; EO: essential oil.

**Table 3:** Destruction effect of different EOs alone and in combination with oxacillin on mature biofilm.

		EO mg/mL		Oxacillin µg/mL		Synergism			
Strains	Essential Oil	sMIC_50_^a^	%Destr. ± SD ^b^	sMIC_50_^c^	% Destr. ± SD ^d^	AB ug/mL ^e^	EO mg/mL ^f^	AB ± EO% Destr. ± SD ^g^	FICI
*E. faecalis ATCC 29212*	*Cinnammonum zeylanicum*	50.4	52.5 ± 0.70	128	66.0 ± 0.19	4.0	2.5	56.6 ± 1.00	0.08
*S.aureus Ig22*	*Cinnammonum zeylanicum*	6.3	52.1 ± 0.70	64	59.1 ± 0.40	2.0	0.3	52.2 ± 0.70	0.08
*S.epidermidis IG4*	*Cinnammonum zeylanicum*	12.6	60.0 ± 1.00	256	64.8 ± 0.09	7.7	0.6	64.3 ± 0.04	0.08
*S. aureus ATCC 29213*	*Cinnammonum zeylanicum*	12.6	48.0 ± 1.00	256	47.8 ± 0.50	7.7	0.6	67.2 ± 0.32	0.08
*E. faecalis ATCC 29212*	*Mentha piperita*	11.3	50.0 ± 1.00	128	66.0 ± 0.18	8.0	0.6	45.2 ± 0.90	0.11
*S. aureus Ig22*	*Mentha piperita*	11.3	51.1 ± 0.60	64	59.1 ± 0.50	2.0	2.3	59.0 ± 0.88	0.08
*S. epidermidis IG4*	*Mentha piperita*	45.5	44.8 ± 0.70	256	64.8 ± 1.00	7.7	2.3	67.6 ± 0.50	0.08
*S. aureus ATCC 29213*	*Mentha piperita*	22.8	45.1 ± 1.00	256	47.8 ± 0.70	15.4	1.1	61.9 ± 1.00	0.11
*E. faecalis ATCC 29212*	*Origanum vulgare*	5.5	49.2 ± 0.60	128	66.0 ± 0.05	4.0	0.3	51.9 ± 1.00	0.08
*S.aureus Ig22*	*Origanum vulgare*	11.0	57.3 ± 1.00	64	59.1 ± 0.39	2.0	0.6	61.7 ± 1.00	0.23
*S. epidermidis IG4*	*Origanum vulgare*	5.8	61.9 ± 0.60	256	64.8 ± 0.60	7.7	0.3	53.3 ± 0.77	0.08
*S. aureus ATCC 29213*	*Origanum vulgare*	5.8	56.8 ± 1.00	256	47.8 ± 1.00	15.4	0.3	52.4 ± 0.35	0.11
*E. faecalis ATCC 29212*	*Thymus vulgaris*	21.7	47.8 ± 0.80	128	66.0 ± 1.00	4.0	1.1	58.1 ± 0.69	0.08
*S. aureus Ig22*	*Thymus vulgaris*	43.5	50.5 ± 0.50	64	59.1 ± 0.13	2.0	2.2	55.4 ± 0.65	0.08
*S. epidermidis IG4*	*Thymus vulgaris*	10.9	54.1 ± 0.30	256	64.8 ± 0.20	30.7	0.5	55.0 ± 0.40	0.17
*S. aureus ATCC 29213*	*Thymus vulgaris*	10.9	59.4 ± 0.40	256	47.8 ± 0.90	30.7	0.5	66.1 ± 1.00	0.17

^a^: sMIC50: sessile minimal inhibitory concentration 50; ^b^: % biofilm destruction ± standard deviation; ^c^: concentration of the antibiotic in the combination; ^d^: Concentration of the EO in the combination; ^e^: concentration of antibiotic in the mixture; ^f^: concentration of essential oil in the mixture; ^g^: biofilm combination mixture inhibition rate; FICI: fractional inhibitory concentration; AB: antibiotic; EO: essential oil.

**Table 4:** Desctruction effect of different EOs alone and in combination with norfloxacin on mature biofilm.

		EO mg/mL		Norfloxacin µg/mL		Synergism			
Strains	Essential Oil	sMIC_50_^a^	% Destr. ± SD ^b^	sMIC_50_^c^	% Destr. ± SD ^d^	AB ug/mL ^e^	EO mg/mL ^f^	AB ± EO% Destr. ± SD ^g^	FICI
*E. faecalis ATCC 29212*	*Cinnammonum zeylanicum*	50.4	52.5 ± 0.70	256	49.2 ± 0.90	8.0	2.5	53.2 ± 1.00	0.08
*S. aureus Ig22*	*Cinnammonum zeylanicum*	6.3	52.1 ± 0.70	512	37.0 ± 0.39	16.0	0.3	60.2 ± 0.77	0.08
*S. epidermidis IG4*	*Cinnammonum zeylanicum*	12.6	60.0 ± 1.00	64	56.0 ± 0.50	2.0	0.6	64.5 ± 0.04	0.08
*S. aureus ATCC 29213*	*Cinnammonum zeylanicum*	12.6	48.0 ± 1.00	256	52.2 ± 1.00	8.0	0.6	66.9 ± 0.90	0.08
*E. faecalis ATCC 29212*	*Mentha piperita*	11.4	50.0 ± 1.00	256	49.2 ± 0.80	16.0	0.6	53.5 ± 0.90	0.11
*S. aureus Ig22*	*Mentha piperita*	11.4	51.1 ± 0.60	512	37.0 ± 0.10	16.0	2.3	47.7 ± 0.12	0.23
*S. epidermidis IG4*	*Mentha piperita*	45.5	44.8 ± 0.70	64	56.0 ± 0.59	2.0	2.3	58.9 ± 0.90	0.08
*S. aureus ATCC 29213*	*Mentha piperita*	22.7	45.1 ± 1.00	256	52.2 ± 0.70	8.0	1.1	50.1 ± 1.00	0.08
*E. faecalis ATCC 29212*	*Origanum vulgare*	5.5	49.2 ± 0.60	256	49.2 ± 0.70	8.0	0.3	53.2 ± 0.20	0.08
*S. aureus Ig22*	*Origanum vulgare*	11.0	57.3 ± 1.00	512	37.0 ± 0.88	32.0	0.6	47.7 ± 0.55	0.11
*S. epidermidis IG4*	*Origanum vulgare*	5.7	61.9 ± 0.60	64	56.0 ± 1.00	2.0	0.3	59.4 ± 0.90	0.08
*S. aureus ATCC 29213*	*Origanum vulgare*	5.7	56.8 ± 1.00	256	52.2 ± 0.05	32.0	0.3	55.6 ± 0.32	0.18
*E. faecalis ATCC 29212*	*Thymus vulgaris*	21.7	47.8 ± 0.80	256	49.2 ± 0.77	8.0	1.1	65.7 ± 0.80	0.08
*S. aureus Ig22*	*Thymus vulgaris*	43.5	50.5 ± 0.50	512	37.0 ± 0.90	16.0	2.2	63.9 ± 0.80	0.08
*S. epidermidis IG4*	*Thymus vulgaris*	10.9	54.1 ± 0.30	64	56.0 ± 0.33	4.0	0.5	52.4 ± 1.00	0.11
*S. aureus ATCC 29213*	*Thymus vulgaris*	10.9	59.4 ± 0.40	256	52.2 ± 0.80	8.0	0.5	58.4 ± 0.90	0.08

^a^: sMIC50: sessile minimal inhibitory concentration 50; ^b^: % biofilm destruction ± standard deviation; ^c^: concentration of the antibiotic in the combination; ^d^: Concentration of the EO in the combination; ^e^: concentration of antibiotic in the mixture; ^f^: concentration of essential oil in the mixture; ^g^: biofilm combination mixture inhibition rate; FICI: fractional inhibitory concentration; AB: antibiotic; EO: essential oil.

## Abbreviations

AMR, Anti-microbic Resistance; EOs, Essential Oils; GC, Gas Chromatography; MS, Mass Spectrometer; SEM, Structural Equation Modeling; LRI, Linear Retention Indices; AI, Arithmetic Index; SI/MS, Similarity Index/Mass Spectra; SD, Standard Deviation; MIC, Minimal Inibitory Concentration; sMIC, sessile Minimal Inibitory Concentration.
